# Searching for New Pharmacological Treatments of Alcohol Use Disorder (AUD): Focus on GLP–1 Receptor Agonists

**DOI:** 10.3390/ijms27104502

**Published:** 2026-05-18

**Authors:** Jolanta B. Zawilska, Ewa Zwierzyńska, Jakub Wojcieszak

**Affiliations:** Department of Pharmacodynamics, Medical University of Lodz, Muszyńskiego 1, 90-151 Lodz, Poland; ewa.zwierzynska@umed.lodz.pl (E.Z.); jakub.wojcieszak@umed.lodz.pl (J.W.)

**Keywords:** glucagon like peptide–1, glucagon like peptide–1 receptor agonists, alcohol use disorder, alcohol dependence, alcohol–associated liver disease, reward pathways

## Abstract

Alcohol use disorder (AUD) remains a crucial public health challenge worldwide. The currently available medications for AUD remain limited in the number and efficacy, meaning that the development of new treatments is of critical importance. Agonists of glucagon–like peptide–1 receptor (GLP–1RAs) have recently received attention as a potential anti–addiction treatment, particularly in AUD. This review presents data from preclinical studies in rodents and non–human primates, registered clinical trials, observational studies, and social media posts, investigating the effects of GLP–1RAs on alcohol–related behaviors and consumption. Several GLP1–RAs and tirzepatide (a dual agonist of GLP–1R and glucose–dependent insulinotropic polypeptide receptor; GIP–R) reduced alcohol consumption and alcohol–seeking behaviors, alcohol–induced locomotor stimulation and memory of alcohol reward, and suppressed relapse drinking in rodents. In addition, they prevent acute alcohol from activating the mesolimbic dopamine system. There are limited human data on the role of the GLP–1 system in AUD. In registered clinical trials, exenatide, semaglutide, and dulaglutide reduced alcohol consumption. Pharmacoepidemiologic studies documented a decreased risk of alcohol–related events in AUD patients using various GLP–1RAs and tirzepatide. Together, existing preclinical and clinical data suggest that GLP–1 is involved in the AUD process and imply the role of GLP1–RAs as a tentative treatment for AUD.

## 1. Introduction

### 1.1. Glucagon–like Peptide–1 (GLP–1)

Glucagon–like peptide–1 (GLP–1) and glucose–dependent insulinotropic polypeptide, also known as gastric inhibitory polypeptide (GIP), belong to incretins—endogenous gut–brain peptides that function both as a hormone and a neuropeptide. GLP–1 is produced by the cleavage of proglucagon by prohormone convertase—peripherally by endocrine L–cells of the intestinal mucosa and α–cells in the pancreatic islets, and centrally by a discrete set of specific neurons within the nucleus tractus solitarius (NTS) of the caudal brainstem. Projections of these neurons are widespread throughout the central nervous system and notably target brain regions associated with reward and reinforcement, such as the nucleus accumbens (NAc), the ventral tegmental area (VTA), and the laterodorsal tegmental area (LDTg). There are different forms of GLP–1: full–length GLP–1 (1–37) or GLP–1 (1–36 amide) and two “truncated” forms—GLP–1 (7–36) amide (“amidated GLP–1”) and GLP–1 (7–37) (“glycine–extended GLP–1”). The truncated forms of the peptide, particularly GLP–1 (7–36) amide, dominate in humans. GLP–1 (7–36) amide is converted into an active peptide GLP–1 (7–37) by cleaving a single arginine residue. GLP–1 has a very short half–life, merely a few minutes. In the blood, it is rapidly degraded by the ubiquitous serum enzyme dipeptidyl peptidase IV (DPP–4) to GLP–1 (9–37) and GLP–1 (9–36 amide), low–affinity ligands for GLP–1 receptor (GLP–1R). The DPP–4–generated GLP–1 metabolites, as well as the intact peptides, are rapidly cleared from the circulation via the kidneys (for an excellent review, see [1]). 

**Figure 1:**
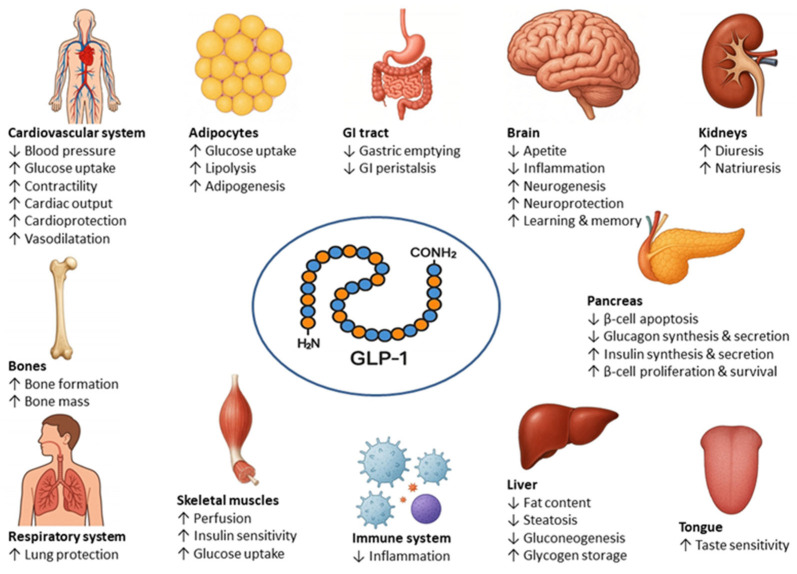
Biological effects of glucagon–like peptide–1 (GLP–1).↓ decrease, ↑ increase; GI—gastrointestinal.

GLP–1 elicits its functions by binding to and activating specific membrane–bound receptors—GLP–1Rs, which belong to a B family of G–protein–coupled receptors (GPCR). GLP–1Rs are widely distributed throughout the body, including the pancreas (β–cells), the kidney and the lung (smooth muscle cells in the walls of arteries and arterioles), the heart (myocytes of the sinoatrial node), the gastrointestinal tract, components of the immune system, such as macrophages, lymphocytes, and invariant natural killer T cells; and the peripheral and central nervous system. In the rodent brain, the highest density of GLP–1Rs was found in the hippocampus, amygdala, and cerebral cortex, structures primarily responsible for memory and cognitive function. Expression of GLP–1Rs was also demonstrated in the brainstem, the limbic system, and in key brain areas involved in motivated behaviors, reward, and addiction (reviewed in [2]). In most regions, GLP–1Rs primarily co–localize with GABAergic neurons, except in the hippocampus, where they are co–expressed with glutamatergic neurons [3]. In the human brain, the highest expression of *GLP1R* mRNA was found in the frontal cortex, followed by the hippocampus, while the orbitofrontal cortex and cerebellum had negligible expression [4]. The best–known activity of GLP–1 is regulation of glucose homeostasis by stimulation of nutrient–induced insulin release from β–cells of the pancreas. In addition, the peptide inhibits glucagon release from pancreatic α–cells, promotes slow stomach emptying, thereby giving the feeling of fullness, and regulates appetite and food intake via appetite– and reward–related areas of the brain. Accumulating data indicate that GLP–1 is also involved in other processes, including, among others, regulation of lipid metabolism, inflammation, blood pressure and heart rate, renal function, learning and memory, and reward behavior. GLP–1 also has cardio– and neuroprotective effects [1] (Figure 1).

### 1.2. Agonists of GLP–1 Receptor (GLP–1RAs)

Due to its very short plasma half–life, GLP–1 has a limited therapeutic utility. To counteract this short duration of GLP–1, extensive work aimed at modifying the structure of the native peptide has been conducted, leading to the development of synthetic compounds that have a partial amino acid sequence homology with the endogenous GLP–1 and act as GLP–1R agonists (GLP–1RAs). These compounds, unlike the endogenous GLP–1, are not easily degraded by DPP–4, resulting in longer half–lives and sustained pharmacological activity to exert therapeutic effects. A range of GLP–1RAs has been formulated, each exhibiting distinct pharmacokinetic characteristics. GLP–1RAs were initially used in patients with type 2 diabetes (T2DM) with expanding clinical indications.

Table 1 presents GLP–1RAs approved by the United States Food and Drug Administration (FDA) for treatment of T2DM and chronic weight management—overweight and obesity. At present, clinically available GLP–1RAs include short–acting (exenatide, a synthetic variant of exendin–4 naturally occurring in the venom of *Gila monster* lizard; injected twice a day), intermediate–acting (liraglutide, injected daily), and long–acting (semaglutide and dulaglutide; administered weekly) medicines. More recently, multi–agonist incretin therapies have expanded the GLP–1 paradigm toward greater weight and metabolic benefits. The first medicine in this group is tirzepatide, which exerts its therapeutic effects through dual agonism of the GLP–1R and GIPR [5].

**Figure 2:**
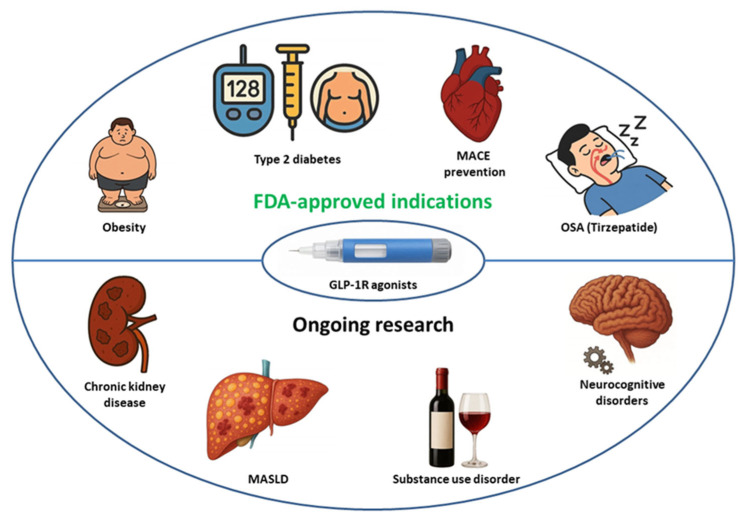
Approved indications for GLP–1R agonists and directions of ongoing research. MACE—major adverse cardiovascular events, MASLD—metabolic dysfunction–associated steatotic liver disease, OSA—obstructive sleep apnea.

Because of the poor bioavailability of peptide drugs, GLP–1RAs are administered as subcutaneous injections, except for semaglutide, which is also given orally. The therapeutic potential of GLP–1 and its analogs extends beyond approved indications, including T2DM, obesity management, obstructive sleep apnea, and prevention of major adverse cardiovascular events (MACE). Emerging evidence suggests an expanding role of GLP–1RAs in the management of chronic kidney disease, metabolic dysfunction–associated steatohepatitis, and neurocognitive disorders [2,4,5,6,7,8,9,10,11] (Figure 2).

### 1.3. Current Pharmacological Treatments for Alcohol and Other Substance Use Disorders (ASUDs)

Alcohol and other substance use disorders (ASUDs) are prevalent, chronic, and relapsing diseases associated with high morbidity and mortality. Treatments for ASUDs include pharmacological and psychosocial interventions. The FDA has approved acamprosate, disulfiram, and naltrexone for alcohol use disorder (AUD), buprenorphine, methadone, and naltrexone for opioid use disorder (OUD), bupropion, varenicline, and nicotine replacement therapies for tobacco use disorder. The European Medicines Agency (EMA) also approved nalmefene for the treatment of alcohol dependence syndrome in adults. Notably, both the FDA and the EMA have not approved any medications for the treatment of psychostimulant or cannabis use disorders [12]. Drugs registered for AUD differ in their mechanisms of action and specific usage restrictions. Naltrexone and nalmefene block µ–opioid receptors, leading to a reduction of dopamine release within the mesolimbic reward pathway. Thereby, they attenuate the rewarding effects of alcohol in patients whose alcohol consumption remains associated with activation of the brain reward circuitry. Disulfiram works primarily by irreversibly inhibiting aldehyde dehydrogenase, an enzyme catalyzing breakdown of acetaldehyde, a toxic byproduct of alcohol, into acetate. A rapid, 5– to 10–fold increase in blood levels of acetaldehyde results in highly unpleasant symptoms (nausea, sweating, headache, hypotension, and heart palpitations) that deter drinking. Because the inhibition is irreversible, the effect can last for 1–2 weeks until new enzyme molecules are synthesized by the liver. The exact mechanism of acamprosate is not fully understood, but it is believed to modulate glutamatergic and GABAergic neurotransmission, which helps reduce craving in patients who are abstinent [13]. Due to the limitations presented above, the discovery and development of new treatments for AUD remains a pressing challenge.

## 2. GLP–1RAs and Alcohol

AUD remains a major public health challenge worldwide [14,15]. At present, drugs approved by the FDA and EMA can help manage AUD by reducing consumption and relapse risk, but their overall effectiveness remains unsatisfactory [16]. The limited efficacy of current pharmacological treatments for AUD underscores the necessity for novel therapeutic approaches. Several lines of evidence, mostly from preclinical experiments but also from preliminary human studies, point to GLP–1RAs as potentially promising new agents for the pharmacological treatment of AUD. Along this line, effects of GLP–1RAs on alcohol use have so far been the most investigated of all addictive substances.

### 2.1. Preclinical Studies

In animal studies, acute and chronic treatments with GLP–1RAs, as well as systemic and central (into selected brain structures) routes of administration, have been used. Most studies tested the GLP–1R agonist exendin–4 (Ex–4), a 39–amino acid peptide derived from the venom of the Heloderma lizard, which shares 53% homology with the native human GLP–1 [5]. Other GLP–1RAs, including exenatide, dulaglutide, liraglutide, and semaglutide, have also been examined. Behavioral studies are aimed to analyze effects of GLP–1RAs on: (1) alcohol intake and drinking pattern using various protocols: 10 to 12 weeks of intermittent access 20% alcohol two–bottle–choice drinking paradigm, intravenous– and oral alcohol self–administration; (2) changes in rewarding effects of alcohol and memory consolidation of a reward—in conditioned place preference (CPP) paradigm, a standard preclinical behavioral test where a stimulus (alcohol) is paired with a context or environment; (3) alcohol–induced locomotor stimulation, and (4) relapse drinking and alcohol withdrawal–induced anxiety–like behavior. In addition, effects of GLP–1RAs on alcohol–induced dopamine release in NAc are assessed using the microdialysis technique in freely moving animals [17].

Several factors contribute to the complexity of AUD. Among them, crucial roles are played by escalated alcohol consumption over time, motivation to consume alcohol, and relapse drinking, which is commonly observed after periods of abstinence [18]. Effects of GLP–1RAs on these factors were addressed by several research groups. Behavioral studies using the two–bottle–choice drinking paradigm and the operant self–administration model demonstrated that both acute and repeated treatment of rodents (rats and mice) with Ex–4 [19,20,21,22,23,24], liraglutide [25,26], dulaglutide [27], and semaglutide [26,28,29] reduce alcohol consumption and preference in a dose–dependent manner. Liraglutide and semaglutide also significantly reduced alcohol intake in alcohol–preferring non–human primates, blue vervet monkeys, without affecting water intake [30,31]. Furthermore, the abilities of alcohol to promote hyperlocomotion, dopamine release in the NAc, and reward in the conditioned place preference paradigm are all suppressed by GLP–1RAs. In mice, systemic administration of Ex–4 not only lowered intake of alcohol but also reduced the number of drinking bouts and increased the time to the first drink [22], suggesting a modulatory action on the drinking pattern. On a similar note, semaglutide lowered binge alcohol drinking in mice and rats [29]. Using the alcohol deprivation model, systemic injection of Ex–4 prevented relapse drinking in male rats [25] and in socially housed male mice [22]. Similarly, liraglutide suppressed relapse drinking in male mice [32], and semaglutide in rats of both sexes [33]. During alcohol withdrawal, mice displayed anxiety symptoms, which were alleviated by systemic injection of liraglutide [32].

Using ^125^I–labeled GLP–1Ras, Salameh et al. evaluated brain uptake pharmacokinetic parameters of nine synthetic peptides in mice. They found that systematically injected acetylated GLP–1 analogs, semaglutide and liraglutide, do not cross the blood–brain barrier (BBB) [34]. Since in preclinical studies, semaglutide and liraglutide injected *sc* or *iv* ameliorate many pathological processes within the brain, including effects of various drugs of abuse, this observation was an unexpected and puzzling one. At present, the question of how these two GLP–1RAs, and likely more, that do not freely cross the BBB can reach their target sites within the brain remains an open one. Among potential hypotheses, the following deserve attention: (1) brain uptake of GLP–1 analogs occurs independent of the BBB; (2) some GLP–1Rs–expressing neurons are in or adjacent to circumventricular regions that lack a BBB and thus can sense peripherally circulating signals and drugs that do not freely cross the BBB; (3) toxic compounds, such as for example alcohol, or pathological processes may disrupt the integrity of the BBB, hence facilitating penetration of drugs into the brain. In support of the latter hypothesis is the demonstration of fluorescent–labeled semaglutide presence in the NAc of alcohol–drinking rats [33].

To understand neuroanatomical circuits involved in GLP–1RAs–evoked changes in alcohol–mediated behaviors, the acute effects of Ex–4 injected directly into selected brain regions were examined. Alcohol consumption was markedly decreased by Ex–4 injected into the VTA, the NAc, the dorsomedial hippocampus, and the lateral hypothalamus. On the contrary, no changes were observed when the drug was injected into the arcuate nucleus and the paraventricular nucleus of the hypothalamus, as well as the basolateral amygdala [35]. When compared to vehicle treatment, intra–VTA Ex–4 infusion significantly reduced alcohol self–administration, an effect that was particularly prominent in high alcohol drinkers. However, VTA Ex–4 did not reduce the reacquisition of alcohol self–administration after extinction nor the motivation to obtain alcohol [36]. Also in mice, the behavioral effects of Ex–4 varied depending on the brain structure examined. Injection of Ex–4 into the NAc shell decreased alcohol intake, blocked alcohol–induced locomotor stimulation, and memory of alcohol reward in the CPP test. Infusion of Ex–4 into LDTg attenuated alcohol–induced locomotor stimulation and reduced alcohol intake, but did not affect memory of alcohol reward. Ex–4 infused into the posterior VTA prevented alcohol–induced locomotor stimulation, but did not modulate CPP–dependent alcohol memory nor alcohol intake. When Ex–4 was injected into the anterior VTA or caudate–putamen, no changes in the tested alcohol–induced behaviors were observed [37,38]. Microinjection of Ex–4 into the NTS, the brain structure where GLP–1 is produced and from which the GLP–1–containing neurons project to areas of reward [17,39,40], blocked the alcohol–induced locomotor stimulation, dopamine release in the NAc, and reward–dependent memory retrieval in the CPP paradigm in mice [37,38]. Likewise, microinjection of Ex–4 into the NTS reduced alcohol intake in rats consuming high amounts of alcohol for 12 weeks. Local infusion into the NTS of exendin–3(9–39) (Ex–9), a selective GLP–1R antagonist, prevented the ability of systemic Ex–4 to block the alcohol–induced locomotor stimulation in mice [37]. Effects of local Ex–4 infusion varied between animals consuming high and low amounts of alcohol. Injection of Ex–4 into the NAc shell or the LDTg potently reduced a 24–h alcohol intake in high– but not in low–alcohol consuming rats [41].

Along this line, Ex–4 infused into the dorsal lateral septum (LS) reduced ethanol self–administration in male rats consuming high amounts of alcohol, but not in low–consuming males or females, regardless of their alcohol response levels [40]. In experiments performed on male mice, systemic administration of Ex–4 blocked the ethanol–induced locomotor stimulation. This effect was not observed in animals treated with intra–LS Ex–9 before systemic Ex–4 administration. Infusion of Ex–4 into the LS reduced alcohol intake, stimulation of locomotor activity, and alcohol–related CPP. On the other hand, alcohol consumption was elevated by intra–LS Ex–9. In male and female rats, intra–LS Ex–4 significantly reduced alcohol intake and alcohol–evoked increase in dopamine release in the NAc shell. Further studies on rats examined the correlation between alcohol consumption and expression of GLP–1R in the LS. Alcohol–preferring male rats exhibited increased GLP–1R levels in the LS but not in the VTA or the LDTg. Of note, GLP–1R expression in the LS correlated with alcohol intake in male but not female rats, an observation suggesting sex–specific effects of the long–term alcohol exposure. Electrophysiological experiments performed on LS slices from alcohol–consuming male and female rats demonstrated that Ex–4 suppresses population spike amplitude in a process involving GABA–A receptors [42].

Vallöf et al. also examined the effects of long–term alcohol consumption on the expression of *Glp1r* gene in the rat prefrontal cortex, the amygdala, the hippocampus, the VTA, the NAc, and the striatum. The *Glp1r* expression in NAc was elevated in high– compared to low alcohol–consuming rats. Furthermore, the expression level of *Glp1r* was positively correlated with alcohol intake. In contrast, no significant differences in *Glp1r* expression in other examined brain structures were found. As GLP–1 is produced by posttranslational processing of the product from the preproglucagon gene (*GCG*), expression of this gene in the aforementioned brain areas was evaluated in high– versus low alcohol–consuming rats. Expression levels of *GCG* in the prefrontal cortex are elevated in high– compared to low alcohol–consuming rats, and there is a positive correlation between *GCG* expression in the prefrontal and increased alcohol intake [41].

To define the molecular basis underlying GLP–1RAs–induced changes in alcohol–related responses, effects of acute systemic administration of semaglutide followed by alcohol injection on in vivo release of dopamine and dopamine metabolites—3,4–dihydroxyphenylacetic acid (DOPAC) and homovanillic acid (HVA)—and on gene expression of enzymes metabolizing dopamine—monoaminoxidase (MAO) and catechol–O–methyl transferase (COMT) in the NAc shell were examined in male mice. Semaglutide did not affect dopamine levels in the NAc shell but lowered the release of this neurotransmitter after alcohol administration. Interestingly, levels of DOPAC and HVA in the NAc shell were higher in male mice treated with the combination of semaglutide and alcohol compared to those treated with alcohol only, an observation indicating that semaglutide enhances the metabolism of dopamine when alcohol is present. Consistent with this, the NAc expression of *Maoa* and *Comt* but not *Maob* genes was higher in animals treated with both semaglutide and alcohol compared to those treated with alcohol only [33].

At a sub–morphological level, effects of liraglutide on alcohol–induced changes in synaptic morphology and levels of synaptic transport–related proteins were examined in the medial prefrontal cortex and the hippocampus of mice. Alcohol consumption led to a reduction in dendritic spine density in both brain structures, which was restored to normal levels by liraglutide. Furthermore, liraglutide increased the levels of synaptic transport–related proteins in mice subjected to alcohol withdrawal [32].

Very recently Edvardsson and coworkers [43] published results of their extensive work on effects of tirzepatide on alcohol intake and alcohol–related responses in rodents. In rats tirzepatide dose–dependently reduced voluntary alcohol consumption in the intermittent access two–bottle choice model, prevented binge alcohol drinking (drinking in the dark paradigm), and entirely blocked relapse–like drinking following forced abstinence. In mice, tirzepatide effectively attenuated the rewarding properties of alcohol, measured through the locomotor stimulation, CPP, and dopamine release in the NAc. An important added value of these studies comes from electrophysiological and proteomic experiments. In electrophysiological experiments, alcohol–naïve mice were acutely treated with tirzepatide. After 24 h, ex vivo neuronal activity with evoked synaptic responses was assessed in slices of the medial prefrontal cortex, the dorsomedial and dorsolateral striatum, the NAc core and shell, and the LS.

Among the structures examined, significant effects of tirzepatide—sustained synaptic depression—were observed only in LS slices. Proteomic analysis of the LS using brain samples from alcohol–consuming male rats receiving repeated tirzepatide demonstrated that, compared to vehicle, 35 proteins were upregulated and 16 proteins downregulated. Among proteins affected by tirzepatide are those involved in neurotransmission, neuroinflammation, and, as previously found, linked to alcohol consumption. Interestingly, significant differences between tirzepatide and vehicle groups were shown for chromatin regulatory proteins: down–regulation of ataxin–3, and up–regulation of histone H1–0, histone H1–4, POU domain class 2 transcription factor 1, dual specificity protein phosphatase 12, histone H2A–1A, chromobox protein homologue 7, histone H3–3B, high mobility group nucleosome–binding domain–containing protein 2, PIH1 domain–containing protein 1, and aprataxin [43].

Overall, a growing body of preclinical evidence indicates that activation of GLP–1Rs suppresses alcohol–seeking behaviors, motivation to consume alcohol, and amount of alcohol intake, as well as relapse drinking in rodents. Moreover, abstinence symptoms experienced during alcohol withdrawal are attenuated by activation of the GLP–1R pathway. On a similar note, stimulation of GLP–1Rs in brain areas involved in reward processing modulates these alcohol–related responses. Taken together, the preclinical findings suggest that GLP–1RAs may represent a novel avenue in pharmacotherapies for AUD, potentially addressing both drinking behavior and the physiological consequences of alcohol consumption that likely contribute to poor treatment outcomes and relapse vulnerability.

### 2.2. Human Data

#### 2.2.1. GLP1R Gene and Alcohol Use

Despite growing data from animal research, clinical evidence on the role of the GLP–1 system in alcohol use and alcohol–related outcomes is scarce. A few studies have investigated the relationship between GLP–1 and GLP–1Rs levels and AUD in humans. In heavy–drinking individuals with AUD, acute administration of alcohol in variable or fixed doses orally or intravenously resulted in a significant reduction of blood GLP–1 concentration. Analysis of *GLP1R* mRNA in post–mortem brain tissues (the amygdala, the VTA, the NAc, the hippocampus, and the prefrontal cortex) demonstrated significantly higher levels in the hippocampus of individuals with AUD compared to controls [44]. Studies on genetic variation of the *GLP1R* gene found that two missense single–nucleotide polymorphisms (SNPs), namely rs6923761 and rs1042044, that result in amino–acid substitutions (rs6923761: glycine to serine at position 168, rs1042044: phenylalanine to leucine at position 260), are associated with the severity of alcohol use [44,45]. The rs6923761 allele was associated with a higher risk of AUD, greater alcohol intake, and breath alcohol measures in the intravenous alcohol self–administration experiment [45]. Neuroimaging studies on interaction between genetic variation of the GLP–1R and severity of alcohol use on a resting–state brain functional connectivity found stronger within–network connectivity in the anterior salience network, the default mode network, the visuospatial network and the basal ganglia network among individuals with high versus low severity of alcohol use (as indicated by AUDIT score), in the groups carrying the risk alleles for each of the two SNPs [44].

#### 2.2.2. Effects of GLP–1RAs on Alcohol Use by Humans

There are four basic sources of information on the effects of GLP–1RAs on alcohol use by humans: randomized clinical trials (RCTs) investigating the role of GLP–1RAs in AUD, clinical evidence, pharmacoepidemiologic studies (real–world data, observational studies), and social media posts.

##### Randomized Clinical Trials

RCTs are still in early stages. As of now, results of only three RCTs have been published. The trials were conducted in outpatient settings with participants diagnosed with AUD, and involved exenatide [46], semaglutide [47], and dulaglutide [48]. There are nine registered clinical trials evaluating the effect of semaglutide in AUD (NCT06015893, NCT05891587, NCT05520775, NCT05892432, NCT05895643, NCT07223983, NCT07218354). Additional drugs under investigation include tirzepatide (NCT06727331, NCT06939088, NCT07046819), and two novel dual agonists of GLP–1R and glucagon receptor: pemvidutide (NCT06987513) and mazdutide (NCT06817356) (for more details, see [49]. The first RCT investigating the effects of GLP–1RA, exenatide (Bydureon, 2 mg *sc* once a week for 26 weeks), was performed by Klausen and coworkers [46] on a group of 127 treatment–seeking AUD patients who had a minimum of 5 heavy drinking days in the past 30 days. After a 6–month follow–up, a number of heavy drinking days and total alcohol intake were evaluated. A total of 58 patients completed the trial. Although exenatide did not significantly reduce the number of heavy drinking days compared with placebo, in a subgroup of obese patients with a BMI higher than 30, it reduced heavy drinking days and total alcohol intake. In those with a BMI under 25, an opposite effect was found—treatment with exenatide increased the number of heavy drinking days, but did not change the total alcohol intake. In the studied group of patients, functional MRI (fMRI) and a single–photon emission computed tomography (SPECT) images were performed at baseline and at week 26. At week 26, during the fMRI spatial working memory test, reduced cue reactivity in the dorsal prefrontal cortex was observed in the exenatide group compared with the placebo group. Moreover, cue–induced activity in the ventral striatum was significantly lower in the exenatide group than in the placebo group. However, in the dorsal striatum and in the putamen, no significant differences were observed. Exenatide lowered dopamine transporter availability (evaluated via SPECT) in the striatum, the caudate, and the putamen [46].

In a phase 2, double–blind, randomized, parallel–arm trial involving 9 weeks of outpatient treatment, the effects of semaglutide on alcohol consumption were evaluated in 48 individuals (NCT05520775). Participants received semaglutide (0.25 mg/week *sc* for 4 weeks, 0.5 mg/week *sc* for 4 weeks, and then 1.0 mg *sc* for 1 week) or placebo at weekly clinic visits. Most patients had a BMI above 30 or between 25.0 and 29.9. Low–dose semaglutide reduced the amount of alcohol consumed during a post–treatment laboratory self–administration task. Treatment with semaglutide significantly reduced drinks per drinking day and weekly alcohol craving, an observation indicating that the drug may lead to greater reductions in heavy drinking over time relative to placebo. In a sample of individuals with current cigarette use, semaglutide treatment predicted greater relative reductions in the number of cigarettes smoked per day. In addition to beneficial effects on AUD parameters, semaglutide also caused a significant reduction in body weight of participants [47].

In another clinical trial (NCT05895643), treatment–seeking patients with moderate to severe alcohol use disorder and comorbid obesity were treated for 26 weeks with either semaglutide (2·4 mg *sc*) or placebo (saline *sc*). Treatment with semaglutide resulted in a significant reduction in heavy drinking days and substantial effects on multiple secondary alcohol–related and somatic outcomes, such as reduction of bodyweight, BMI, and waist circumference. Notably, authors found that reduced body weight correlated with reduced heavy–drinking days [50]. However, caloric intake was not assessed in this study, thus it cannot be determined whether the reduction of body weight was due to the effects of semaglutide on appetite and metabolism or due to reduced consumption of calorie–rich alcoholic beverages.

Probst and coworkers conducted a double–blind, randomized, placebo–controlled trial evaluating dulaglutide (0.75 mg/0.5 mL *sc*. in the first week, increased to 1.5 mg/0.5 mL in the following weeks until the end of treatment) as a therapy for smoking cessation and found that participants treated for smoking cessation drink significantly less alcohol after 12 weeks of treatment with dulaglutide compared with placebo, without correlation to smoking status [48].

##### Clinical Evidence

Fourteen patients with hazardous alcohol drinking problems were prescribed a GLP–1RAs/GIP–RA for treatment of overweight or obesity: one liraglutide, eight semaglutide, and five tirzepatide. Significant reductions not only in BMI but also in Alcohol Use Disorders Identification Test (AUDIT) scores over time were observed, with patients from the very high AUDIT group reporting more pronounced reductions in AUDIT scores compared with those in the high AUDIT group [51]. In a retrospective chart review of six patients, treatment with semaglutide for weight loss also led to reductions in AUD symptomatology [52].

##### Real–World Evidence

Pharmacoepidemiologic studies using electronic health records and national registries have provided real–world evidence on alcohol–related outcomes in patients treated with GLP1–RAs, often in populations with co–morbid T2DM or obesity.

In a large–scale (227,866 individuals) cohort study, using nationwide registry data for Swedish residents with a diagnosis of AUD, semaglutide and liraglutide, but not other GLP–1RAs, were associated with a markedly reduced risk of AUD– and SUD–related hospitalizations as well as somatic hospitalizations [53]. Wium–Andersen and coworkers [54] utilizing data from nationwide registers in the Danish population, examined whether the use of GLP–1RAs and DPP–4 inhibitors was associated with a decreased risk of alcohol–related events. Using GLP–1RAs was linked with a lower risk of subsequent alcohol–related events (i.e., hospital contacts with a main diagnosis of AUD, registered treatments for AUD, purchase of alcohol, or alcohol withdrawal pharmacotherapy) than using DPP–4 inhibitors. However, this association was observed only during the first 3 months of treatment. The authors of the study concluded that “GLP–1RAs did not appear to be viable alternatives to existing treatments for AUD” [54]. In Ireland, analysis of electronic health records of 262 obese patients treated with liraglutide or semaglutide demonstrated a significant reduction in alcohol intake (mean decrease of 7.1 units/week) with a weak positive correlation between alcohol reduction and weight loss. In high consumers, the alcohol intake was reduced by 68% [55]. In a retrospective cohort study of patients diagnosed concurrently with AUD and metabolic dysfunction, including obesity (BMI > 25) and/or a history of T2DM, semaglutide or tirzepatide therapy was associated with lower 1–year AUD relapse [56]. In a case series of six patients with positive AUD screenings who were treated with semaglutide for weight loss, a significant improvement in AUD symptoms (mean decrease of 9.5 points) was found [52].

There are five published reports from observational studies conducted in the United States. Analysis of data from over 817,000 patients with a documented history of AUD from the Oracle Cerner Real–World Data revealed that prescriptions of tirzepatide and GLP–1RAs—albiglutide, dulaglutide, exenatide, liraglutide, lixisenatide, and semaglutide—were linked to a 50% decrease in alcohol intoxication events compared to those without a prescription for these medicines. Overall, when stratified by T2D, obesity, and T2D and obesity, an incident alcohol intoxication rate was, respectively, 49%, 42%, and 42% lower, compared to those found in patients without GIP/GLP–1RAs prescriptions [57]. Wang et al. conducted a large retrospective cohort study using electronic health records from the TriNetX Platform. Of the 83,825 obese patients without a prior diagnosis of AUD, 45,797 received prescriptions for semaglutide, and 38,028 received non–GLP–1RA anti–obesity drugs, topiramate, or naltrexone. Semaglutide, but not other analyzed drugs, reduced the risk of incident AUD and AUD relapse in patients with obesity or T2DM [58]. Recently, Gougol and coworkers conducted a retrospective cohort study of 1946 patients at Stanford Health Care with a concurrent diagnosis of AUD and metabolic dysfunction (MetD), including obesity and/or a history of T2DM with HbA1c > 5.7. Of them, 274 were treated with GLP–1RAs, 1272 with naltrexone, 232 with acamprosate, and 168 with disulfiram. GLP–1RA therapy was associated with lower 1–year AUD relapse, greater BMI reduction, and HbA1c improvement [56]. Using real–world electronic health record data from the U.S. Department of Veterans Affairs, associations between the use of GLP–1RAs (liraglutide, semaglutide, dulaglutide, exenatide, and albiglutide) or DPP–4 inhibitors (alogliptin, saxagliptin, sitagliptin, and linagliptin) and changes in alcohol use were evaluated using Alcohol Use Disorders Identification Test–Consumption (AUDIT–C) scores. Patients treated with GLP–1RAs reported a greater reduction in AUDIT–C scores than those receiving DPP–4 inhibitors and unexposed individuals. There were no differences between the last two groups [59]. In a retrospective nested case–control study within a well–defined cohort of adults enrolled in the All of Us Research Program, the association between GLP–1RAs treatment and incident substance use disorders, including, among others, AUD, was evaluated. The AUD cohort included 11,326 patients and 11,326 controls. Exposure to GLP–1RAs was associated with a 74% reduction in the odds of AUD diagnosis. The strongest association was observed with semaglutide (85% reduction in AUD odds), followed by dulaglutide, exenatide, and liraglutide [60].

Quddos and coworkers, after analyzing social media discussions centered around GLP–1R/GIP–R agonists and alcohol use (see below), recruited 153 participants to examine changes in alcohol consumption in subjects with diabetes or obesity treated for ≥30 days with semaglutide (56) or tirzepatide (50) and those not using any medication for these diseases (control group). Individuals on semaglutide or tirzepatide had, on average, significantly fewer drinks and declared reduced drinking both on weekdays and weekends when compared to the control group. Furthermore, the odds of binge drinking were significantly lower in the medication groups as compared to the control group [61].

##### Analysis of Social Media Posts

Despite its limitations, analysis of social platforms/forums is a feasible approach for exploring the multifaceted aspects of drug use/misuse patterns.

Three recent studies analyzed social media posts, mainly from the Reddit platform, but also TikTok and YouTube, uploaded by individuals taking GLP–1RAs for diabetes and/or obesity [61,62,63]. Across all user comments, the most prevalent themes were related to self–reported reduced drinking quantity and loss of interest/desire to drink. In one of the analyses, 29.75% of alcohol–related comments clearly stated a cessation of the intake of alcohol, and 14.42% reduced use following the start of the GLP–1RAs use [63]. Other frequently reported themes included reduction of cravings or urges to drink, enhancement of aversive effects of alcohol, and changes in subjective responses and sensitivity to effects of alcohol both decreased (typically reported as reductions in the hedonic/rewarding effect of alcohol), and increased (e.g., stronger intoxicating effects) [61,62,63].

## 3. GLP–1RAs and Alcohol–Associated Liver Disease (ALD)

Alcohol–associated liver disease (ALD) is a frequent complication of excessive alcohol intake (>350 g/wk for females and 420 g/wk for males) that can encompass a spectrum of conditions from fatty liver (steatosis), inflammation (alcohol–associated hepatitis), and cirrhosis that may lead to hepatocellular carcinoma [64]. Importantly, ALD is one of the leading preventable causes of illness and death from liver diseases worldwide [65]. Several preclinical and clinical studies have shown the efficacy of GLP–1RAs in the treatment of obesity, metabolism–associated fatty liver disease (MAFLD), and nonalcoholic steatohepatitis (NASH) [66]. However, to date, only a very few studies have addressed the efficacy of GLP–1RAs as a treatment modality for ALD.

### 3.1. Preclinical Studies

In basic research, an experimental NIAAA model of ALD was used. In this model, animals are chronically orally fed ad libitum with the Lieber–DeCarli ethanol liquid diet, followed by a single binge ethanol dose. The first study examining whether a GLP–1RA could reduce ALD pathogenesis was conducted in rats by Mahalingam and coworkers [67]. They found that treatment with Ex–4 significantly reduced ethanol intake and improved alcohol–induced alterations in liver/body weight and adipose/body weight ratio, impairments in glucose tolerance, carbohydrate metabolism, hepatic insulin sensitivity, and serum levels. In ethanol–fed rats, Ex–4 treatment reduced elevated levels of serum alanine transferase (ALT) —a marker of liver injury, reduced hepatic fat accumulation by decreasing fatty acid uptake and synthesis, and increased hepatic fatty acid oxidation. These effects of Ex–4 treatment led to a reduction in indices of the chronic alcohol–induced hepatic steatosis [67].

Another study using an experimental animal model of ALD was conducted in mice. Treatment of mice with semaglutide resulted in a reduced intake of both control liquid and ethanol diets, and a corresponding reduction of body weight in both the control and ethanol groups. Compared with the control diet, the animals fed with the ethanol diet exhibited hepatic steatosis indices that were significantly reduced by semaglutide. The reduction in steatosis was accompanied by suppressed expression of hepatic genes for *de novo* lipogenesis (*Acc1*, *Srebp1c*, and *Fasn*), for oxidative stress (*Nrf2*, *Nox2*), and inflammation (*Mpo*, *Il–6*, *Il–12*). Furthermore, immunostaining experiments demonstrated a significant increase in hepatic lipid peroxidation in mice on ethanol, which was suppressed by semaglutide treatment [68].

### 3.2. Human Data

Rashid and coworkers [69], utilizing data from the IBM MarketScan, analyzed the impact of GLP–1RAs on adverse liver outcomes among patients with ALD and T2DM. Non–GLP–1RAs patients had a significantly higher incidence of hepatic decompensation vs. patients taking GLP–1RAs. In contrast, no difference was found in the incidence of portal hypertension or liver transplantation. The authors conclude that GLP–1RAs may potentially mitigate the risk of adverse liver outcomes among patients with ALD. Future randomized controlled trials are needed to prove the role of these medications in patients with ALD and T2DM.

Using the TriNetX Research Network, retrospective studies comparing the effects of GLP–1RAs versus DPP–4 inhibitors were conducted on two cohorts of patients: (1) patients with T2DM and AUD but without ALD, (2) patients with T2DM and established ALD. Patients who suffered from AUD and were on GLP–1RAs had a 38% lower risk of developing alcohol–related histological liver lesions in comparison to those treated with DPP–4 inhibitors. In the ALD cohort, the use of GLP–1RAs was associated with a significantly lower risk of hepatic decompensation than DPP–4 inhibitor use. In both cohorts, using GLP–1RAs was associated with markedly reduced all–cause mortality compared with DPP–4 inhibitors [70].

A randomized clinical trial in which patients with AUD and comorbid obesity were treated with semaglutide (NCT05895643) demonstrated a significant reduction in plasma γ–glutamyl transferase (GTT) activity compared to placebo. Plasma GTT activity is commonly utilized as a marker of liver damage, often secondary to alcohol consumption. Therefore, findings of the mentioned clinical trial suggest a potential hepatoprotective property of GLP–R agonists, especially if the liver damage is due to alcohol [50].

## 4. Conclusions

AUD remains a major global problem affecting millions of people. Despite decades of research, the currently available medications for AUD remain limited in the number and efficacy, meaning that the discovery and development of new treatments for this disease are of critical importance. One of the emerging and potentially promising pharmacotherapeutic approaches for AUD is the modulation of the GLP–1 system via GLP–1RAs. GLP–1RAs, originally approved for treating T2D and obesity, have recently received attention as a potential anti–addiction treatment, particularly in AUD. Studies in rodents and non–human primates demonstrated that various GLP–1RAs decrease alcohol intake, attenuate alcohol–seeking behavior, reduce motivation to consume alcohol, and prevent relapse drinking by lowering alcohol–induced reward. Preclinical and clinical data also suggest that GLP–1RAs may exert hepatoprotective effects in ALD by reducing hepatic steatosis, oxidative stress, and inflammation. Despite growing and promising data from preclinical studies, clinical evidence on the role of the GLP–1 system in alcohol use and alcohol–related outcomes is scarce. In particular, at present, the number of RCTs involving GLP–1RAs is very limited. Along this line, large–scale RCTs that include diverse populations are essential to determine not only the effectiveness of these drugs, but also the underlying mechanism(s) of action. Although molecular mechanisms by which GLP1–RAs impact alcohol drinking and related behavior are yet to be fully elucidated, accumulating data point out that they differ from those described for AUD registered drugs (see Section 1.3). Data from preclinical and clinical studies indicate that interaction with central GLP–1Rs, associated with the reduction of alcohol cue–induced activation of the reward system, is crucial for lowering alcohol intake [46,71]. However, as recently emphasized by Haass–Koffler [72], an interplay between the central effects of GLP–1RAs and their peripheral mechanisms that modulate metabolism and gastric motility cannot be ruled out. One of the major peripheral effects of GLP–1RAs is slowing down the gastric emptying, which delays alcohol absorption from the small intestine, leading to lower peak concentrations. Via this mechanism, GLP–1RAs may reduce the acute effects of alcohol and lower its reinforcing properties. Additionally, delayed gastric emptying stimulates vagal signaling, which in turn influences brain regions related to the reward system and impulse control [72].

Finally, it should be emphasized that there is a critical need for future studies aimed at careful investigation of the safety and tolerability of GLP–1Ras, as well as factors like dosage, duration of treatment, and potential rebound effects upon treatment cessation. In addition, as the effects of incretin combinations on alcohol–related effects remain largely unknown, further work should address this issue.

## Figures and Tables

**Table 1:** FDA–Approved Indications for GLP–1 and Dual GIP/GLP–1 Receptor Agonists (based on [6]).

Agent (Trade Name)	Half–Life Time	T2DM (Year of Approval)	Obesity (Year of Approval)	Cardiovascular Risk Reduction (Year of Approval)
Exenatide IR (Byetta^®^)	2–3 h	Yes ^#^ (2005)	No	No
Exenatide ER (Bydureon^®^)	2 weeks	Yes (2012)	No	No
Lixisenatide (Adlyxin^®^)	2–4 h	Yes (2016)	No	No
Dulaglutide (Trulicity^®^)	5 days	Yes (2014)	No	Yes * (2020)
Liraglutide (Victoza^®^, Saxenda^®^)	11–15 h	Yes (2010)	Yes (2014)	Yes ** (2017)
Semaglutide (Ozempic^®^, Wegovy^®^)	160 h	Yes (2017)	Yes (2021)	Yes ** (2020)
Oral semaglutide (Rybelsus^®^)	165–184 h	Yes (2019)	No	No
Tirzepatide (Mounjaro^®^, Zepbound^®^)	19–25 h	Yes (2022)	Yes (2023)	No

Tirzepatide is a dual GIP and GLP–1 receptor agonist. ^#^ The first GLP–1RA approved for T2DM. * Approved to reduce the risk of major adverse cardiovascular events (cardiovascular death, nonfatal myocardial infarction, or nonfatal stroke) in adults with T2DM and established cardiovascular disease or multiple cardiovascular risk factors. ** Approved to reduce the risk of major adverse cardiovascular events (cardiovascular death, nonfatal myocardial infarction, or nonfatal stroke) in adults with T2DM and established cardiovascular disease.

## Data Availability

No new data were created or analyzed in this study. Data sharing is not applicable to this article.

## Abbreviations

The following abbreviations are used in this manuscript:

ALD	alcohol–associated liver disease
ALT	alanine aminotransferase
ASUDs	alcohol and other substance use disorders
AUD	alcohol use disorder
AUDIT–C	Alcohol Use Disorders Identification Test–Consumption
BBB	blood–brain barrier
BMI	body mass index
COMT	catechol–O–methyl transferase
CPP	conditioned place preference
DOPAC	3,4–dihydroxyphenylacetic acid
DPP–4	dipeptidyl peptidase IV
EMA	European Medicines Agency
Ex–4	exendin–4
Ex–9	exendin–3(9–39)
FDA	Food and Drug Administration
fMRI	functional magnetic resonance imaging
GI	gastrointestinal
GCG	preproglucagon gene
GIP	glucose–dependent insulinotropic polypeptide
GIP–R	glucose–dependent insulinotropic polypeptide receptor
GLP–1	glucagon–like peptide–1
GLP–1R	glucagon like peptide–1 receptor
GLP–1RAs	agonists of glucagon like peptide–1 receptor
GPCR	G–protein–coupled receptors
GTT	plasma γ–glutamyl transferase
HVA	homovanillic acid
LDTg	laterodorsal tegmental area
LS	lateral septum
MAO	monoaminoxidase
MACE	major adverse cardiovascular events
MASLD	metabolic dysfunction–associated steatotic liver disease
MetD	metabolic dysfunction
NAc	nucleus accumbens
NASH	nonalcoholic steatohepatitis
NTS	nucleus tractus solitaries
OSA	obstructive sleep apnea
OUD	opioid use disorder
RCTs	randomized clinical trials
SNPs	single nucleotide polymorphisms
SPECT	single–photon emission computed tomography
SUD	substance use disorder
T2DM	type 2 diabetes
VTA	ventral tegmental area
